# AI-generated explanations in kidney transplantation: accuracy vs. readability and implications for patient education

**DOI:** 10.3389/frai.2026.1806516

**Published:** 2026-03-11

**Authors:** Oscar A. Garcia Valencia, Charat Thongprayoon, Jing Miao, Iasmina M. Craici, Wisit Cheungpasitporn

**Affiliations:** Department of Internal Medicine, Division of Nephrology and Hypertension, Mayo Clinic, Rochester, MN, United States

**Keywords:** AI-generated explanations, artificial intelligence, kidney care, kidney transplantation, large language models, nephrology, patient education, readability assessment

## Abstract

**Background:**

Effective patient education is critical for informed decision-making and adherence in kidney transplantation. Generative artificial intelligence (AI), particularly large language models (LLMs), has the potential to enhance patient education in kidney transplantation; however, its factual accuracy and readability remain incompletely characterized.

**Methods:**

We evaluated the performance of the GPT-5.1 (2025) model in generating plain-language explanations for 100 clinically relevant kidney transplantation terms. Explanations were generated using a standardized prompt (first round) and a revised prompt explicitly requesting an eighth-grade reading level or lower (second round). Accuracy was assessed by expert reviewers using a 5-point Likert scale, while readability was evaluated using the Flesch Reading Ease (higher score indicated easier readability of the text) and Flesch–Kincaid Grade Level (higher score indicated higher education level required to understand the text) score. The study was conducted in November 2025.

**Results:**

All AI-generated explanations demonstrated high accuracy, with no clinically significant errors. In the first round, the mean Flesch Reading Ease score was 23.6 ± 23.4, indicating very difficult readability, and 46% of explanations required a college-level reading ability (mean Flesch–Kincaid Grade Level 13.4 ± 4.8). Following prompt revision, readability improved substantially. The mean Flesch Reading Ease score increased to 62.4 ± 7.5, corresponding to standard readability, and all explanations were written at a middle school level or below (mean Flesch–Kincaid Grade Level 6.3 ± 1.1).

**Conclusion:**

GPT-5.1 generated highly accurate explanations of kidney transplantation terms across prompting strategies. Explicit readability-focused prompting substantially improved readability without compromising accuracy, underscoring the importance of prompt design when deploying LLMs for patient-centered education in transplantation.

## Introduction

Kidney transplantation remains the preferred treatment for end-stage kidney disease (ESKD), offering superior survival and quality of life compared to dialysis (Zhu et al., 2025; Chadban et al., 2020; Puttarajappa et al., 2021). However, long-term graft survival is influenced not only by surgical and immunological factors but also by patients’ ability to understand, engage with, and adhere to complex post-transplant care regimens (Wekerle et al., 2017; Strohmaier et al., 2022). Effective management requires a thorough understanding of immunosuppressive therapy, strict adherence to prescribed medications, and early recognition of transplant-specific complications (Zhu et al., 2025; Chadban et al., 2020; Puttarajappa et al., 2021). Deficits in health literacy pose a major barrier to these processes, contributing to medication nonadherence, increased rejection rates, and poorer clinical outcomes (Maasdam et al., 2022; Vidnes et al., 2024).

Recent advances in artificial intelligence (AI), particularly the development of large language models (LLMs), have created new opportunities to support patient-centered education (Aydin et al., 2024; Thirunavukarasu et al., 2023; Cheungpasitporn et al., 2026; Tangri et al., 2025). LLMs can generate plain-language medical explanations and adapt content to different literacy levels, potentially enabling scalable, personalized educational support for transplant recipients (Will et al., 2025; Ellison et al., 2025). By simplifying complex transplant-related concepts, AI-generated explanations may help bridge persistent knowledge gaps that limit patient understanding and engagement (Demirbaş et al., 2025; Umman et al., 2025; Xue et al., 2024). At the same time, the use of AI-generated content in high-stakes clinical contexts such as transplantation raises critical concerns regarding factual accuracy, readability, and reliability, as even subtle misinformation may have serious clinical consequences (Nasra et al., 2025; Bedi et al., 2025; Moëll and Sand Aronsson, 2025; Deeb et al., 2024).

Health literacy plays a pivotal role in determining healthcare outcomes (Paasche-Orlow, 2011; Magnani et al., 2018). The National Assessment of Adult Literacy (NAAL) has reported that only 12% of U.S. adults demonstrate proficient health literacy, while approximately 22% have basic literacy, and 14% have below-basic health literacy (Magnani et al., 2018; Wittink and Oosterhaven, 2018). In parallel, the average U.S. adult reads at approximately an eighth-grade level, underscoring the difficulty of producing educational materials that are both clinically accurate and broadly comprehensible (Armache et al., 2024; Karakurt et al., 2025; Hyvert et al., 2023). Striking an appropriate balance between accessibility and medical precision is therefore essential. Overly simplified explanations risk omitting clinically relevant details, whereas excessively technical descriptions may overwhelm or disengage patients with limited health literacy, ultimately undermining shared decision-making and adherence (Agarwala et al., 2023; Dennison Himmelfarb et al., 2023).

Despite growing interest in AI-driven patient education tools, systematic evaluations of LLM-generated educational content in kidney transplantation remain limited (Will et al., 2025; Demirbaş et al., 2025; Tian Tran et al., 2024; Zhan et al., 2025; Garcia Valencia et al., 2023). In particular, the extent to which prompt design influences the balance between readability and clinical accuracy has not been well characterized (Will et al., 2025; Demirbaş et al., 2025; Bedi et al., 2025; Tian Tran et al., 2024; Zhan et al., 2025). Accordingly, this study evaluates the ability of a contemporary LLM to generate plain-language explanations for 100 clinically relevant kidney transplantation terms. Expert clinician assessments of factual accuracy and objective readability metrics were used to determine whether explicit readability-focused prompting can enhance accessibility without compromising clinical accuracy, thereby informing the responsible deployment of LLMs for patient education in transplantation (Table 1).

**Table 1:** Accuracy, and readability of AI-generated explanations for kidney transplant-related terms.

Metric	1st round	2nd round
Accuracy		
- Highly inaccurate - Somewhat inaccurate - Neutral - Mostly accurate - Highly accurate	0 (0) 0 (0) 0 (0) 3 (3) 97 (97)	0 (0) 0 (0) 0 (0) 4 (4) 96 (96)
Flesch reading ease readability score	23.6 ± 23.4	62.4 ± 7.5
- Very easy - Easy - Fairly easy - Standard - Fairly difficult - Difficult - Very difficult	0 (0) 1 (1) 5 (5) 5 (5) 5 (5) 19 (19) 65 (65)	0 (0) 1 (1) 15 (15) 45 (45) 37 (37) 2 (2) 0 (0)
Flesch–Kincaid grade level	13.4 ± 4.8	6.3 ± 1.1
- Elementary school - Middle school - High school - College or above	6 (6) 12 (12) 36 (36) 46 (46)	37 (37) 63 (63) 0 (0) 0 (0)

## Methods

### Study design and overview

This study adhered to the TRIPOD-LLM (Transparent Reporting of a Multivariable Prediction Model for Individual Prognosis or Diagnosis using Large Language Models) guidelines to ensure a systematic and reproducible evaluation of AI-generated medical explanations (Gallifant et al., 2025). We assessed the ability of the GPT-5.1 (2025) model to explain 100 clinically relevant kidney transplantation terms using independent expert evaluations and objective readability metrics. The study was conducted in November 2025.

### Selection of kidney transplantation terms

The evaluated content consisted of 100 predefined kidney transplantation–related terms rather than complex clinical cases or diagnostic scenarios. These terms represent commonly encountered concepts in transplant counseling, immunosuppressive management, rejection, complications, and long-term graft care. The objective was to assess the model’s ability to generate accurate and readable explanations of discrete medical concepts frequently discussed during patient education, rather than to evaluate performance in case-based clinical reasoning or individualized decision-making. We identified 100 commonly encountered kidney transplant-related terms (Supplementary Table S1), ensuring clinical relevance and coverage of key concepts in patient education, immunosuppression management, and transplant complications. Terms were selected from transplant guidelines (e.g., KDIGO, AST, UNOS), frequently used terminology in patient education materials, and common patient inquiries during transplant clinic visits (Zhu et al., 2025; Chadban et al., 2020; Puttarajappa et al., 2021).

### AI-generated explanations

At the time of the study (November 2025), GPT-5.1 was the most recent large language model available. Each term was individually queried using a standardized prompting strategy to generate plain-language explanations intended for patients. Prompts emphasized clarity, patient-centered language, and medical accuracy. A second round of queries was subsequently conducted using a revised prompt that explicitly requested explanations written at an eighth-grade reading level or lower.

First prompt: “*Please provide a brief explanation of each of these terms for patients, and keep each description very accurate but short.”*

Revised prompt: “*Please modify the text to make it easier to understand for someone who reads at or below an 8th grade level.”*

### Accuracy and readability assessment

Two investigators (OAGV and CT) jointly reviewed each AI-generated explanation and assigned accuracy ratings by consensus using a 5-point Likert scale. Factual accuracy and clinical relevance was evaluated using a 5-point Likert scale (Storino et al., 2016; Daraz et al., 2019; Joshi et al., 2015):

1 = Highly inaccurate (contains major errors or misleading content).
2 = Somewhat inaccurate (minor factual errors or misinterpretations).
3 = Neutral (correct but lacks clarity or omits key details).
4 = Mostly accurate (clinically sound with minor phrasing issues).
5 = Highly accurate (fully correct, well-structured, and relevant).

The readability of AI-generated explanation was assessed using the Flesch Reading Ease and Flesch–Kincaid Grade level (Solnyshkina et al., 2017; Jindal and MacDermid, 2017). The Flesch Reading Ease and Flesch–Kincaid Grade scores are calculated based on average number of words per sentence and average number of syllables per word. The Flesch Reading Ease score is calculated with the following formula: 206.835–1.015 x (total words / total sentences) - 84.6 x (total syllables / total words). The higher Flesch Reading Ease score indicates easier readability and can be interpreted as below (Solnyshkina et al., 2017; Jindal and MacDermid, 2017).

90–100: very easy to read.
80–89: easy to read.
70–79: fairly easy to read.
60–69: standard.
50–59: fairly difficult to read.
30–49: difficult to read.
0–29: very difficult to read.

The Flesch–Kincaid Grade score is a readability test that estimates the education level required to understand the text. The Flesch–Kincaid Grade score is calculated with the following formula: 0.39 × (total words / total sentences) + 11.8 × (total syllables / total words) − 15.59. The higher Flesch–Kincaid Grade score indicates the higher level of education generally required to understand the text, and can be interpreted as below (Solnyshkina et al., 2017; Jindal and MacDermid, 2017).

1–5: elementary school.
6–8: middle school.
9–12: high school.
≥13: college or above.

The study was conducted twice, one week apart, to evaluate the consistency of the accuracy and readability of AI-generated explanations.

### Statistical analysis

The accuracy of AI-generated explanations, assessed using a 5-point Likert scale, was summarized as counts with corresponding percentages. Readability, evaluated using the Flesch Reading Ease and Flesch–Kincaid Grade Level metrics, was summarized as means with standard deviations and as frequencies with percentages, as appropriate. Because the same 100 terms were evaluated in both prompting rounds, readability outcomes were analyzed using paired t-tests to compare mean Flesch Reading Ease and Flesch–Kincaid Grade Level scores between rounds. A two-sided *p*-value <0.05 was considered statistically significant. All statistical analyses were performed using JMP statistical software, version 17 (SAS Institute, Cary, NC).

## Results

### Accuracy of AI-generated explanations

AI-generated explanations demonstrated high factual accuracy. In the first round, 97% of explanations were rated as highly accurate, with the remaining 3% rated as mostly accurate. Similarly, in the second round, 96% of explanations were rated as highly accurate and 4% as mostly accurate. No explanations in either round were rated as neutral or inaccurate.

### Readability of AI-generated explanations

In the first round, the mean Flesch Reading Ease score was 23.6 ± 23.4, indicating that the AI-generated explanations were, on average, very difficult to read. Consistent with this finding, most explanations fell within the difficult (19%) or very difficult (65%) categories, and nearly half (46%) required a college-level reading ability based on the Flesch–Kincaid Grade Level.

Following implementation of a revised prompt explicitly requesting explanations written at an eighth-grade reading level or lower, readability improved substantially. The mean Flesch Reading Ease score increased to 62.4 ± 7.5, corresponding to standard readability. All explanations in the second round were written at a middle (63%) or elementary (37%) school level, with no explanations requiring high school or college-level reading ability. Paired analysis confirmed that readability improved significantly after prompt revision (Flesch Reading Ease: paired *p* < 0.001; Flesch–Kincaid Grade Level: paired *p* < 0.001).

## Discussion

This study demonstrates that a contemporary LLM can generate educational content on kidney transplantation with consistently high clinical accuracy across a broad range of commonly used terms. Across both prompting strategies, no clinically significant inaccuracies were identified, underscoring the potential reliability of LLM-generated explanations when applied to transplant-related patient education.

The high proportion of explanations rated as highly accurate may in part reflect the structured and concept-based nature of the evaluated content. The study focused on predefined transplant-related terminology rather than complex clinical cases, individualized management decisions, or ambiguous real-world scenarios. Explaining discrete medical terms may represent a lower cognitive and inferential demand for large language models compared with tasks requiring clinical reasoning, contextual integration, or risk stratification. Accordingly, the observed accuracy should not be interpreted as evidence of uniform reliability across all transplant-related applications. Future studies should extend evaluation frameworks to include case-based reasoning, complex clinical counseling scenarios, and edge cases in which factual precision and contextual judgment are more rigorously tested.

The principal finding of this study is not a trade-off between accuracy and readability, but rather the critical role of prompt design in shaping readability while preserving clinical accuracy (Ellison et al., 2025; Demirbaş et al., 2025; Tian Tran et al., 2024; Halawani et al., 2024; Halawani et al., 2024). When generated using a generic prompt, explanations were factually correct but linguistically complex, with readability levels exceeding those appropriate for the average adult reader. Following readability-focused prompting, readability improved substantially, with all explanations meeting middle school-level thresholds, while accuracy remained unchanged. These findings highlight prompt engineering as a key determinant of usability for AI-generated patient education materials (Ellison et al., 2025; Akkan and Seyyar, 2025; Eid et al., 2024; Kufta and Djalilian, 2025).

Our results align with prior studies demonstrating that LLMs often produce technically accurate but overly complex medical explanations when no readability constraints are specified (Aydin et al., 2024; Thirunavukarasu et al., 2023; Cao et al., 2024; Amin et al., 2024; Li et al., 2025). However, this study extends the existing literature by showing that readability deficits are not intrinsic to LLM-generated content and can be effectively mitigated through targeted prompt modification (Will et al., 2025; Ellison et al., 2025; Halawani et al., 2024; Eid et al., 2024; Srinivasan et al., 2024). Unlike earlier reports suggesting an inherent tension between accuracy and accessibility, our findings indicate that both can be achieved simultaneously when readability is explicitly incorporated into prompt design (Kufta and Djalilian, 2025; Nassar et al., 2025; Patel et al., 2024; Dihan et al., 2024; Dihan et al., 2025; Daram et al., 2025).

The observed improvements in Flesch Reading Ease and Flesch–Kincaid Grade Level scores following prompt revision are clinically meaningful. Prior work has demonstrated that educational materials exceeding recommended readability thresholds are associated with poorer comprehension, reduced adherence, and diminished shared decision-making, particularly among patients with limited health literacy (Magnani et al., 2018; Armache et al., 2024; Okuhara et al., 2025; Rustomji et al., 2025). By achieving standard readability without loss of accuracy, LLM-generated explanations may better support patient understanding in transplantation, a field characterized by complex terminology and lifelong self-management demands (Wittink and Oosterhaven, 2018; Dennison Himmelfarb et al., 2023; White-Williams et al., 2020). Importantly, this study shifts the focus from model capability to deployment strategy. While much of the AI literature emphasizes model architecture or training data, our findings suggest that how LLMs are instructed may be as important as which model is used, particularly in patient-facing applications (Abdulnour et al., 2025; Shah et al., 2024; Singhal et al., 2023). This has practical implications for clinicians, educators, and health systems seeking to integrate AI tools into transplant education workflows, as prompt templates can be standardized, audited, and refined without requiring changes to underlying models (Umman et al., 2025; Shah et al., 2023; Peloso et al., 2022).

An important consideration is the dynamic and rapidly evolving nature of LLMs. Model performance may change over time due to updates in training data, alignment strategies, architecture refinements, or deployment configurations. As a result, the accuracy and readability observed in this study reflect the performance of GPT-5.1 at a specific point in time and cannot be assumed to remain static. Continuous validation, version transparency, and periodic re-evaluation are therefore essential when integrating LLMs into clinical education workflows. Health systems adopting AI-based educational tools should implement structured monitoring processes to ensure sustained factual reliability, readability standards, and alignment with current clinical guidelines. Such safeguards are particularly important in high-stakes domains such as transplantation, where even subtle shifts in content generation may have clinical implications.

Several limitations warrant consideration. Accuracy ratings were assigned by two clinician reviewers using a consensus approach rather than independent scoring; accordingly, inter-rater reliability could not be quantified. Future studies should incorporate independent multi-rater evaluations with prespecified adjudication procedures and reporting of inter-rater agreement to strengthen reproducibility. The analysis was limited to 100 predefined kidney transplantation terms and may not reflect the linguistic and contextual complexity of longitudinal educational materials or real-time clinical conversations (Zhu et al., 2025; Chadban et al., 2020; Puttarajappa et al., 2021). Although standardized readability metrics provide objective and reproducible estimates, they do not directly measure patient comprehension, recall, or decision-making, and they incompletely capture contextual understanding, numeracy, cultural factors, and emotional burdeni (Storino et al., 2016; Daraz et al., 2019; Joshi et al., 2015). Finally, because the evaluation was conducted using a single model and within a specific prompting framework, generalizability to other LLM architectures and deployment environments remains uncertain.

Future research should incorporate patient-centered outcomes, including comprehension testing, satisfaction, and behavioral measures such as medication adherence. In addition, the concentration of ratings in the highest accuracy categories observed in this study may reflect both the factual consistency of the model outputs and the constrained scope of the evaluated content, which consisted of discrete transplant-related terms rather than complex clinical scenarios. The use of a 5-point Likert scale, while commonly applied in similar evaluations, may have limited discriminatory sensitivity in detecting subtle qualitative differences among highly accurate explanations. Future investigations should therefore consider more granular scoring frameworks, independent multi-rater panels, or structured error taxonomies to enhance differentiation among high-performing outputs. Comparative evaluations across multiple LLMs and languages, as well as the integration of multimodal educational tools (for example, visuals, diagrams, and interactive explanations), may further enhance accessibility (Umman et al., 2025; Hamid et al., 2022; Burghall et al., 2023). In addition, embedding readability constraints as default parameters within clinical AI systems could promote safer and more equitable dissemination of transplant education materials (Olmeda Barrientos et al., 2021; Ihsan et al., 2025).

In conclusion, this study demonstrates that LLM-generated explanations of kidney transplantation concepts can achieve both high clinical accuracy and appropriate readability when guided by explicit prompt design. These findings support the responsible use of LLMs as scalable tools for patient education in transplantation, provided that readability is treated as a core design requirement rather than an afterthought. Thoughtful deployment of AI-generated educational content has the potential to improve patient understanding, engagement, and shared decision-making, ultimately contributing to better transplant outcomes.

## Data Availability

The original contributions presented in the study are included in the article/Supplementary material, further inquiries can be directed to the corresponding author/s.
