# Supplementary material for: AI-generated explanations in kidney transplantation: accuracy vs. readability and implications for patient education
Source: Front Artif Intell. 2026 Mar 11;9:1806516. doi: 10.3389/frai.2026.1806516 (PMC13012974; doi:10.3389/frai.2026.1806516)
Supplement: Supplementary file 1 [file Data_Sheet_1.PDF]

Table S1: 100 commonly encountered kidney transplant-related terms

1. Kidney Transplant
2. End-Stage Kidney Disease (ESKD)
3. Dialysis
4. Transplant Candidate
5. Deceased Donor
6. Living Donor
7. Waiting List
8. UNOS Status
9. Preemptive Transplant
10. Retransplantation
11. Expanded Criteria Donor (ECD)
12. Standard Criteria Donor (SCD)
13. Kidney Paired Donation (KPD)
14. Donor Specific Antibody
15. Sensitization
16. Transplant Evaluation
17. Blood Type Matching
18. HLA Typing
19. Crossmatch Test
20. Kidney Allocation Score (KAS)
21. Pre-Transplant Vaccination
22. Psychosocial Assessment
23. Living Donor Workup
24. Medical Non-Adherence
25. Organ Procurement
26. Nephrectomy
27. Anastomosis
28. Warm Ischemia Time (WIT)
29. Cold Ischemia Time (CIT)

30. Native Nephrectomy
31. Immunosuppressants
32. Calcineurin Inhibitors (CNIs)
33. Antiproliferative Agents
34. Steroids
35. mTOR Inhibitors
36. Induction Therapy
37. Anti-Rejection Therapy
38. Antiviral Prophylaxis
39. Antibacterial Prophylaxis
40. Antifungal Prophylaxis
41. Therapeutic Drug Monitoring (TDM)
42. Delayed Graft Function (DGF)
43. Acute Rejection
44. Chronic Rejection
45. Cytomegalovirus (CMV)
46. BK Virus Nephropathy
47. Post-Transplant Diabetes Mellitus (PTDM)
48. Proteinuria
49. Graft Loss
50. Post-Transplant Lymphoproliferative Disorder (PTLD)
51. Recurrent Disease
52. Ureteral Complications in Kidney Transplant
53. Opportunistic Infections in Transplant Recipients
54. Graft Function
55. Immunosuppression Regimen
56. Rejection Workup
57. Allograft Biopsy
58. Therapeutic Drug Levels
59. Adherence

60. Biopsy-Proven Acute Rejection (BPAR)
61. Allograft Dysfunction
62. Graft Survival
63. Banff Classification of Renal Allograft Pathology
64. Acute Tubular Injury (ATI) in Transplants
65. Interstitial Fibrosis and Tubular Atrophy (IFTA)
66. C4d Staining for Antibody-Mediated Rejection
67. Peritubular Capillaritis (PTC)
68. Transplant Glomerulopathy (TG)
69. Donor-Derived Endotheliopathy
70. T-Cell Mediated Rejection (TCMR)
71. Microvascular Inflammation (MVI)
72. Chronic Active Antibody-Mediated Rejection (CAMR)
73. Ischemia-Reperfusion Injury Pathology
74. Subclinical Rejection
75. Thrombotic Microangiopathy (TMA) in Transplants
76. De Novo Immune Complex Glomerulonephritis
77. Focal Segmental Glomerulosclerosis (FSGS) Recurrence
78. Recurrent and De Novo Membranous Nephropathy
79. Lupus Nephritis in Allografts
80. Recurrent IgA Nephropathy in Transplants
81. Calcineurin Inhibitor Toxicity
82. Post-Transplant Thrombotic Thrombocytopenic Purpura (TTP)
83. Recurrent Alport Syndrome Pathology
84. Collapsing Glomerulopathy in Transplants
85. Chronic Graft Glomerulopathy
86. Eosinophilic Infiltrate and Acute Interstitial Nephritis
87. Post-Transplant Thrombus Formation and Infarction
88. C3 Glomerulopathy in Transplants
89. Anti-Glomerular Basement Membrane (Anti-GBM) Disease in Allografts

90. Chronic Calcineurin Inhibitor Nephrotoxicity (Arteriolar Hyalinosis)
91. Donor-Derived Disease Pathology
92. Amyloid Deposition in Allografts
93. Acute Antibody-Mediated Rejection (AAMR)
94. Chronic Allograft Nephropathy (CAN)
95. Hemodialysis Post-Transplant
96. Peritoneal Dialysis Post-Transplant
97. Transplant Infectious Disease
98. Recurrent Glomerulonephritis Post-Transplant
99. Medical Adherence
100. Post-Transplant Monitoring
